# Putative Epigenetic Regulator microRNAs (epi-miRNAs) and Their Predicted Targets in High-Fat Diet-Induced Cardiac Dysfunction: An In Silico Analysis in Obese Rats

**DOI:** 10.3390/ijms26052247

**Published:** 2025-03-03

**Authors:** Márton Pipicz, Gergő Zalán Biró, Márton Richárd Szabó, Ágnes Zvara, Tamás Csont

**Affiliations:** 1 Department of Biochemistry, Albert Szent-Györgyi Medical School, University of Szeged, H-6720 Szeged, Hungary; pipicz.marton@med.u-szeged.hu (M.P.);; 2 Interdisciplinary Centre of Excellence, University of Szeged, H-6720 Szeged, Hungary; 3 Laboratory of Functional Genomics, Core Facility, HUN-REN Biological Research Centre, H-6726 Szeged, Hungary

**Keywords:** high-fat diet, epi-miRNAs, network analysis, cardiac dysfunction, epigenetic regulators, miRNA-mRNA interactions, histone modification, DNA methylation, cardiac remodeling

## Abstract

Obesity-related cardiac dysfunction is a significant global health challenge. High-fat diets (HFDs) are well-established models of obesity. HFD has been reported to induce cardiac dysfunction and alter cardiac miRNA expression, DNA methylation and histone modifications. Nevertheless, it remains unclear whether cardiac miRNAs altered due to HFD target epigenetic regulator enzymes and function as epigenetic regulator miRNAs (epi-miRNAs), thereby contributing to HFD-induced epigenetic changes and cardiac dysfunction. To address this gap in our knowledge, this study aimed to identify putative cardiac epi-miRNAs and their potential epigenetic targets through an in silico analysis of a previously published miRNA dataset from Sprague Dawley rats subjected to HFD. Using two independent databases, miRDB and miRWalk, predicted miRNA-mRNA interactions were analyzed. A total of 71 miRNAs were identified in our present study as putative epi-miRNAs. A total of 34 epi-miRNAs were upregulated (e.g., miR-92b-3p, let-7c-5p, miR-132-3p), and 37 were downregulated (e.g., miR-21-3p, miR-29c-3p, miR-199a-3p) in response to HFD. Epi-miRNAs targeted 81 individual epigenetic regulators (e.g., Dnmt3a, Ezh2, Hdac4, Kdm3a) with 202 possible miRNA–target interactions. Most of the targeted epigenetic regulators were involved in histone modification. An epi-miRNA–target analysis indicated increased DNA methylation and histone acetylation and decreased histone methylation in the hearts of HFD-fed rats. These findings suggest the importance of epi-miRNA-induced epigenetic changes in HFD-related cardiac dysfunction.

## 1. Introduction

Obesity and obesity-related diseases represent a global health problem and significant epidemiological burden [1]. In 2022, the World Health Organization reported that 43% of adults were overweight, and 16% were obese in the European Region [2]. Obesity is associated with hyperlipidemia, diabetes and hyperuricemia, and it increases the risk of cardiovascular diseases e.g., coronary artery diseases or heart failure represented by cardiac dysfunction [3]. Cardiac dysfunction in obesity is characterized by structural and functional changes in the heart, driven by multiple mechanisms, including hemodynamic overload, metabolic alterations and chronic inflammation [4]. These factors contribute to cardiac remodeling and fibrosis [4,5], leading to diastolic and later systolic dysfunction and subsequent heart failure.

Obesity is a complex, multifactorial disease influenced by genetic, environmental and epigenetic factors [1]. Sedentary lifestyle and high-calorie diet are considered as the major drivers of developing obesity [6]. Therefore, diet-induced obesity is the most common model in preclinical obesity research [7]. High-fat diets (HFDs), particularly those combining high fat and high fructose, are effective in inducing obesity, metabolic syndrome and cardiac dysfunction [7,8,9,10]. HFD induces several changes in the heart, including increased oxidative stress, impaired mitochondrial function and alterations in microRNA (miRNA) expression [10], DNA methylation/hydroxymethylation [11] and histone modifications [12,13], potentially linking epigenetic changes to cardiac dysfunction.

miRNAs are small (typically 20–25 nucleotides long), non-coding RNA molecules that regulate gene expression post-transcriptionally [14]. miRNAs target specific mRNAs through binding to complementary sequences of mRNAs, leading to translational repression or degradation of the target mRNAs. Certain miRNAs play crucial roles in cardiac development and function and influence processes such as contractility, hypertrophy, fibrosis and regeneration of the heart [15]. The dysregulation of miRNAs is associated with various cardiac diseases, including cardiomyopathy and heart failure [15,16].

Epigenetic regulator miRNAs or epigenetic microRNAs (epi-miRNAs) are a subclass of miRNAs that play a crucial role in regulating the epigenetic machinery [17,18]. These epi-miRNAs can directly target the mRNA of key enzymatic effectors of DNA methylation or histone modifications such as DNA methyltransferases (Dnmts), histone acetyltransferases (Hats) or deacetylases (Hdacs). Recent research has identified numerous epi-miRNAs in cardiac diseases, including let-7c, mir-9, miR-21-3p and miR-133a, among others [19].

Some studies have shown that HFD induces cardiac dysfunction and leads to changes in cardiac miRNAs, DNA methylation or histone modifications [10,11,12]. Nevertheless, it is still not clear whether the cardiac miRNAs altered due to HFD target epigenetic regulator enzymes and thus play a putative role as epi-miRNAs in HFD-induced epigenetic changes and cardiac dysfunction. To address this question, in the present study we performed an in silico analysis on previously published data to reveal potential cardiac epi-mirRNAs in HFD and to identify their possible epigenetic regulator targets.

## 2. Results

### 2.1. Putative Epi-miRNAs in HFD-Induced Cardiac Dysfunction

Out of the originally reported 94 differently expressed cardiac miRNAs due to HFD [10], 71 miRNAs were identified in our present study as putative epigenetic regulator miRNAs (epi-miRNAs). These epi-miRNAs targeted epigenetic regulator enzymes in both predicted miRNA–target interaction databases (i.e., miRDB and miRWalk, respectively) (Table 1). A total of 34 epi-miRNAs were upregulated, and 37 were downregulated in response to HFD (Table 1). A total of 37 epi-miRNAs (16 upregulated, 21 downregulated) reached the high-threshold predictive limit (i.e., ≥80 target score in miRDB and ≥0.92 binding probability in miRWalk) (Table 1, blue background).

Out of the 71 epi-miRNAs, 15 epi-miRNAs have been identified previously as epi-miRNAs in non-HFD animal or human studies (Table 1), according to the literature. Out of these previously confirmed epi-miRNA–target connections, five interactions were predicted in our analysis as well (miR-26a-5p—Ezh2, let-7d-5p—Kdm3a, mir-199a-3p—Kdm3a, mir-199a-3p—Dnmt3a and miR-29c-3p—Dnmt3a). Our study revealed 56 novel predicted epi-miRNAs and 197 novel possible miRNA–epigenetic regulator interactions (Table 1). A total of 76% of the epi-miRNAs were found to target more than two epigenetic regulators (Table 1). Among the high-threshold predicted miRNA–target interactions, 10 targets have been validated previously (Table S1): miR-31a-5p—Wdr5, miR-22-3p—Phf8, miR-132-3p—Kdm5a (Jarid1a), miR-199a-3p—Kdm3a, miR-92b-3p—Ezh2, miR-26a-5p—Usp3, miR-874-5p—Sirt3, miR-7a-5p—Prkcb, miR-26a-5p—Ezh2, miR-29c-3p—Dnmt3a.

**Figure 1:**
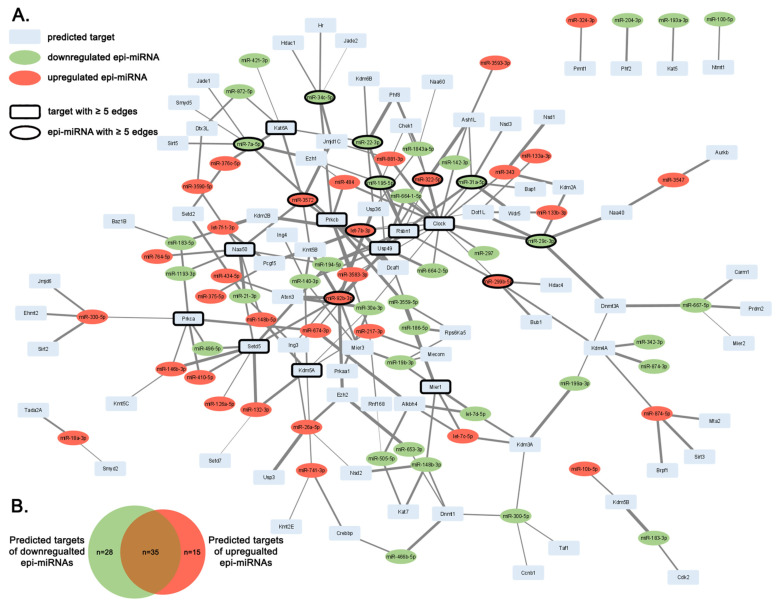
Interaction network of epi-miRNAs and their predicted mRNA targets. (**A**) Upregulated, downregulated epi-miRNAs and targeted mRNAs are indicated in red, green and blue, respectively. Nodes represent epi-miRNAs induced by high-fat diet and putative target mRNAs, while edges symbolize miRNA–target interaction prediction score. Widths of edges are in accordance with cumulative prediction score values. Nodes with at least 5 interactions are highlighted with black border. (**B**) Venn diagram illustrates number of predicted targets of down- and upregulated epi-miRNAs. Green and red region of Venn diagram represents number of predicted targets exclusively regulated by downregulated or upregulated miRNAs, respectively. Brown color represents number of targets that are theoretically modulated by both down- and upregulated miRNAs.

To visualize the complexity of these interactions and reveal possible hubs, an miRNA–target network was constructed as illustrated on Figure 1.

The upregulated miR-92b-3p, let-7b-3p, miR-299b-5p, miR-322-5p and miR-3572 and downregulated miR-31a-5p, miR-140-3p, miR-195-5p, miR-22-3p, miR-29c-3p, miR-34c-5p and miR-7a-5p targeted at least five epigenetic regulators (Table 1, Figure 1). miR-92b-3p has the most targets (seven targets) among the identified epi-miRNAs (Table 1, Figure 1).

### 2.2. Predicted Targets of Epi-miRNAs with Epigenetic Regulator Function

In the present study, 81 epigenetic regulators were predicted as a target of cardiac epi-miRNAs in HFD-induced cardiac dysfunction (Table 2) out of the total 219 epigenetic regulators expressed in healthy heart tissue (Table S2). The number of targets exhibiting a low or medium expression in the heart was nearly equal (Table 2), while highly expressed epigenetic regulators were not detected in this study.

The targeted epigenetic regulators predicted in this study exhibit the following annotated functions (Table 2): DNA-methyltransferase activity (2 targets), DNA demethylase activity (1 target), histone acetyltransferase activity (15 targets), histone deacetylase activity (10 targets), histone methyltransferase activity (22 targets), histone demethylase activity (13 targets), histone ubiquitin ligase activity (3 targets), histone deubiquitinase activity (4 targets) and histone kinase activity (11 targets). There were no predicted epigenetic regulators with histone succinyltransferase activity, histone glutaryltransferase activity, histone butyryltransferase activity or histone phosphatase activity.

**Figure 2:**
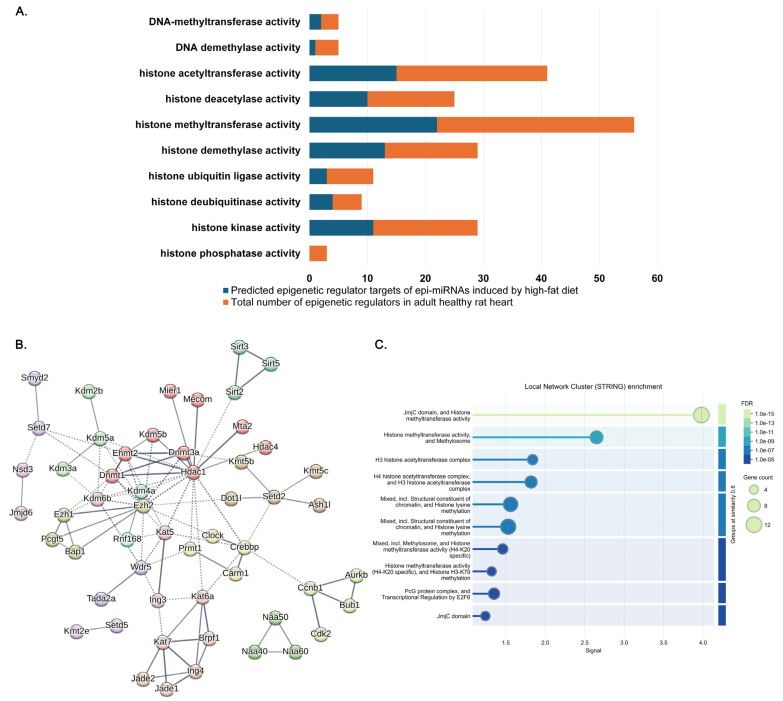
Number of predicted epigenetic regulators of epi-miRNAs induced by a high-fat diet (HFD) and their functional network and enrichment analysis. (**A**) Number of predicted epigenetic regulators targeted by epi-miRNAs under HFD-induced cardiac dysfunction compared to the total number of epigenetic regulators in a given functional category in the adult healthy rat heart. (**B**) Functional protein–protein interaction network and subsequent cluster analysis. The identified epi-miRNA-regulated protein network is visualized according to the Markov Clustering Algorithm using the STRING database and the in-built plugin of Cytoscape v3.9.0. The edges represent functional interactions between nodes. The minimum required interaction score was set to high confidence (0.700). Edges between clusters are indicated by dotted lines. (**C**) Result of Local Network Cluster (STRING) enrichment analysis.

Most of the epigenetic regulators potentially regulated by epi-miRNAs were related to histone modification (Table 2, Figure 2A). Regulators with histone methyltransferase activity (27.2% of the total number of predicted epigenetic regulator targets) or histone acetyltransferase activity (18.5% of the total predicted targets) were the most prevalent among the identified targets (Table 2).

The most regulated target was the clock circadian regulator Clock (also known as Kat13d), regulated by 17 epi-miRNAs (23.9% of total epi-miRNAs) (Table 2, Figure 1). The following epigenetic regulators were also highly targeted by epi-miRNAs: Setd5 (by eight epi-miRNAs), Naa50 and Rsbn (by seven epi-miRNAs), Kdm5a, Mier1, Prkca, Prkcb and Usp49 (by six epi-miRNAs) and Kat6a, targeted by five epi-miRNAs (Table 2, Figure 1).

We found that 29.6% of the identified epigenetic regulator targets in our study were implicated in heart diseases, according to disease ontology (Table 2). The congestive heart failure-associated targets were Dnmt1, Hdac1, Hdac4, Ezh2, Sirt3, Kdm2a, Prkca and Prkcb (9.8% of total targets) (Table 2). The cardiomyopathy-associated targets were Dnmt1, Hdac1, Dot1l, Setd5, Nsd2, Usp3, Bap1, Prkca, Prkcb, Crebbp and Clock: 11 out of 81 (13.6% of total targets) (Table 2). A total of 70.4% of targets have not been annotated to heart disease to date, suggesting novel, unexplored roles in cardiac remodeling triggered by HFD (Table 2).

While 18 putative epigenetic regulator mRNAs were regulated exclusively by upregulated miRNAs (e.g., Brpf1, Hdac4, Sirt 2, Sirt 3, Pcgf5, Bub1), 28 epigenetic regulators were targeted only by downregulated miRNAs (e.g., Dnmt1, Dnmt3a, Hdac1, Sirt5, Bap1, Cdk2) (Table 2, Figure 1B). A total of 35 epigenetic regulators were theoretically modulated by both up- and downregulated miRNAs (e.g., Clock, Crebbp, Kat6a, Mier1, Setd5, Usp49) (Table 2, Figure 1B). Although the total number of epigenetic regulators with DNA-methyltransferase activity was only two, both Dnmt1 and Dnmt3a were targeted by downregulated miRNAs, suggesting the probability of increased methylation activity in cardiac dysfunction (Table 2).

Figure 2A illustrates the number of predicted epigenetic regulators targeted by epi-miRNAs under HFD-induced cardiac dysfunction compared to the total number of epigenetic regulators in a given functional category in the adult healthy rat heart. Substantially differing ratios (percentages) indicate that histone kinase (38%), DNA methyltransferase (40%) and histone deubiquitination (44%) activities seemed to overcome phosphate (0%), DNA demethylase (20%) and histone ubiquitination (27%) activities, respectively. The results also suggest a slight imbalance in histone methylation–demethylation (39% and 45%, respectively) and acetylation–deacetylation (37% and 40%, respectively) (Figure 2A).

A functional network analysis performed on target–target interactions of the epigenetic regulators revealed clusters such as DNA methylation on cytosine and histone deacetylase activity (Figure 2B, red), histone H3 acetylation (Figure 2B, brown), PKMTs methylate histone lysines (Figure 2B, dark gold), histone arginine methylation (Figure 2B, yellow) or the PcG protein complex (Figure 2B, olive). The analysis revealed a few hubs such as Hdac1, Ezh2 or Dnmt1 (Figure 2B).

Local Network Cluster (STRING) analysis revealed significant enrichments (Figure 2C), and the histone methyltransferase activities showed the largest signals, with H4-K20 and H3-K79 specificity. Biological Process (Gene Ontology) enrichment showed that histone H3-K36 methylation, H4-K20 dimethylation and histone H3-S28 phosphorylation had a high strength (Figure S1).

### 2.3. Prediction of Overall Epigenetic Changes in HFD-Induced Cardiac Dysfunction

**Figure 3:**
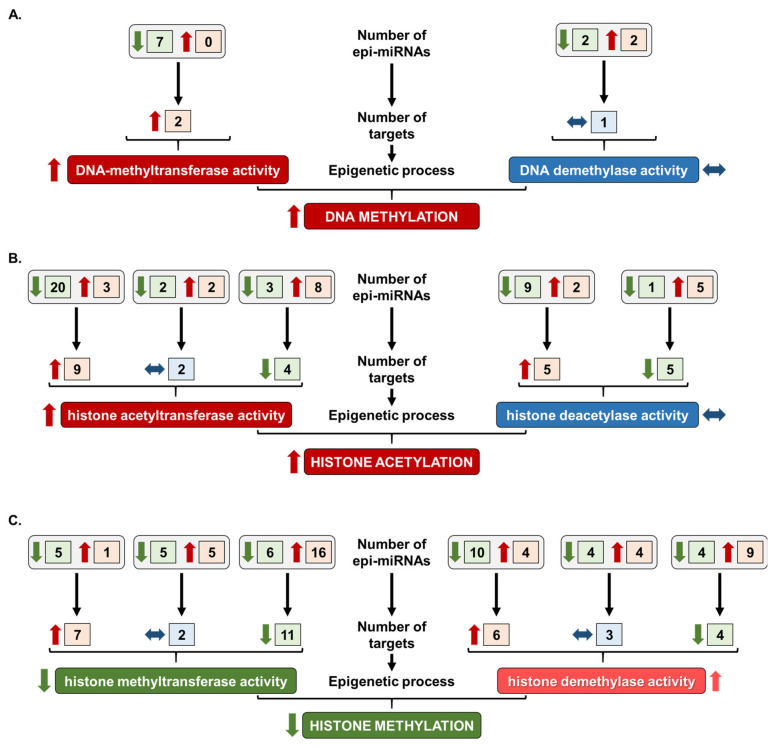
Prediction of the overall epigenetic changes in high-fat diet (HFD)-induced cardiac dysfunction based on the landscape of epi-miRNA alteration due to HFD. Colors and arrows indicate upregulation (red, ↑), slight upregulation (bright red, ↑), downregulation (green, ↓) or no change (blue, <->).

A comprehensive epi-miRNA–target analysis was applied to propose the possible overall epigenetic changes in HFD-induced cardiac remodeling. The analysis involved the number of epi-miRNAs, the direction of the change in epi-miRNAs due to HFD, the number of predicted targets with a given epigenetic regulatory function and the possible change in the targets’ level, based on the alterations in epi-miRNAs. This prediction focused on the most studied epigenetic modifications such as DNA methylation, histone acetylation and histone methylation. The results indicated increased DNA methylation and histone acetylation and decreased histone methylation in the hearts of HFD-fed rats (Figure 3A–C).

## 3. Discussion

In the present study, with the help of bioinformatic analysis tools, we revealed 71 putative epi-miRNAs and 81 individual predicted epigenetic regulator targets in HFD-induced cardiac dysfunction. To the best of our knowledge, this is the first study that identified HFD-induced cardiac miRNAs targeting epigenetic regulators and, based on epi-miRNA alterations, predicted possible changes in the epigenetic landscape of the heart of HFD-fed rats. Moreover, we identified a complex interaction network of epi-miRNAs and their targets, with 197 novel possible miRNA–epigenetic regulator interactions. The subsequent functional analysis of the targets suggested an imbalance in the opposing epigenetic processes, indicating probable changes in cardiac DNA methylation and histone modifications due to HFD. These epigenetic modifications likely contribute to cardiac dysfunction and remodeling, as many of the epi-miRNA-induced epigenetic regulators identified in our study have been previously linked to heart diseases. The epi-miRNAs and their epigenetic regulator counterparts identified in the current study may represent putative targets—following experimental validation—for the development of novel therapeutic approaches against cardiac dysfunction and remodeling in obesity.

Epi-miRNAs are deeply involved in the epigenetic control of various cellular processes [17,19]. These non-coding RNAs can either be influenced by epigenetic mechanisms such as DNA methylation and histone modifications, or they can directly modulate epigenetic changes themselves. A total of 34 of the epi-miRNAs we identified here were upregulated and 37 were downregulated in response to HFD. A few of them have been previously implicated as epi-miRNAs in non-HFD models, mainly in human cancers (see Table 1). Some epi-miRNAs appear to target the same epigenetic regulators, suggesting redundancy in their function. Certain epigenetic regulators are targeted by a broad array of epi-miRNAs, marking them as hubs in their network. The extensive targeting of Clock by both upregulated and downregulated miRNAs introduces a novel perspective on the relationship between circadian rhythm disruption and cardiac dysfunction. This pattern is less explored in the context of HFD-induced cardiac remodeling. The circadian clock of cardiomyocytes has an impact on cardiac physiology and diseases (for a review, please see [39]). Cardiomyocyte-specific CLOCK mutation affected the responsiveness of the heart to hypertrophic stimuli [40] and altered cardiac metabolic adaptation to high fatty acid levels [41]. The bidirectional regulation of certain targets (e.g., Clock, Kat6a and Setd5) by both upregulated and downregulated miRNAs suggests a more complex and dynamic regulatory network than previously reported. This observation could reflect a finely tuned mechanism balancing gene expression in response to HFD-induced metabolic stress.

There are many papers proposing that epigenetic modifications, such as DNA methylation and histone modifications, contribute to the development of cardiac hypertrophy and heart failure [19,42,43].

Interestingly, in the present study both Dnmt1 and Dnmt3a were targeted by downregulated miRNAs, suggesting the probability of increased methylation activity in a chronic stage of HFD-induced cardiac dysfunction. Increased DNMT3A epigenetically silences RASSF1A, which contributes to isoproterenol-induced cardiac fibrosis through the upregulation of ERK1/2 [44]. According to Fang et al., an elevated cAMP level in HL-1 cardiomyocytes results in an increased expression of DNMT3a and global DNA methylation, leading to an increased expression of proteins (e.g., Gata4, Mef2c, Nfatc1, Myh7) related to cardiac hypertrophy [45]. Transcriptional factor Gata4 induces the expression of hypertrophy-responsive genes such as β-myosin heavy chain (Myh7) and natriuretic peptide A [46]. Madsen et al. have demonstrated that DNMT3A knockout human cardiomyocytes are resistant to hypertrophic signaling-induced functional impairment [47]. Several studies reported that DNA methylation inhibitors exhibited a protective effect on cardiac function in in vitro and in vivo models of cardiac hypertrophy [48,49,50]. These results suggest that the hypermethylation of the DNA may contribute to hypertrophic heart disease and cardiac fibrosis.

let-7c-5p, miR-26a-5p, miR-133a-3p, miR-92b-3p epi-miRNAs were upregulated in HFD, and they target Enhancer of zeste homolog 2 (EZH2), as it was validated by previous experiments, suggesting the possibility of EZH2 downregulation in HFD-induced cardiac dysfunction. In accordance, maternal HFD was shown to decrease EZH2 levels in the heart of adult offspring [13]. EZH2 is the catalytic subunit of the polycomb repressive complex 2, which methylates lysine 27 on histone H3, leading to gene repression [51]. It was proposed that HFD-induced EZH2 downregulation leads to the upregulation of proteins (e.g., Isl1, Six1, Mef2c) contributing to the promotion of cardiac fibrosis [13]. Dal-Pra et al. demonstrated that the inhibition of EZH2 in cardiac fibroblasts increased the RNA and protein levels of Tbx5, Hand2 and Gata4 [24]. Paul Delgado-Olguín et al. have suggested that EZH2 may suppress gene expression that promotes cardiac fibrosis [52]. Taken together, these findings may suggest a pathogenic role of the downregulation of EZH2 by epi-miRNAs in HFD that contributes to cardiac fibrosis and dysfunction. Our functional network analysis also identified EZH2 as a potential hub.

The downregulation of let-7d-5p, miR-199a-3p and miR-300-5p in HFD-induced cardiac remodeling may result in the overexpression of the histone demethylase Kdm3a, which is a validated target of let-7d-5p and miR-199a-3p. In transgenic mice with postnatal myocyte-selective KDM3A overexpression exposed to transaortic constriction, a more severe cardiac hypertrophy was observed [53]. KDM3A promoted pro-fibrotic gene expression, activated TIMP1 and resulted in cardiac fibrosis [53]. In hyperglycemia-mediated myocardial injury, it was reported that KDM3A acts through the NFκB pathway, leading to excessive oxidative stress, apoptosis, inflammation and subsequent myocardial injury [54]. These findings suggest that the epi-miRNA-mediated upregulation of KDM3a in HFD may play a pathogenic role in cardiac fibrosis and dysfunction.

DOT1L, which is a highly conserved lysine-specific histone methyltransferase, may also play a crucial role in the development of the heart. According to our study, three epi-miRNAs targeted Dot1l, suggesting the downregulation of DOT1L in HFD-induced cardiac dysfunction. The research of Nguyen et al. demonstrated that the knockout of the Dot1l gene induces severe dilatative cardiomyopathy in mice through dystrophin impairment in cardiac tissue [55].

Our study may have some limitations, similar to all other studies. This study provides insights into the regulatory roles of miRNAs in HFD-induced cardiac dysfunction through in silico predictions. Computational prediction analysis was carried out by complex algorithms incorporating multiple factors to strengthen the reliability of prediction, but this may introduce the risk of false targets. A cardiac mRNA dataset from the same HFD-fed animals would support our miRNA–target predictions. Although many miRNAs are conserved, the translatability of our findings to humans has limitations. Cardiac miRNA profiling was conducted following a 24-week feeding period in Zou’s work [10], without analyzing cell-specific (e.g., cardiomyocyte, fibroblast, endothelial cell) differences. Epi-miRNA changes at this stage may play a role of maintaining cardiac dysfunction and/or contributing to disease progression, or the observed epi-miRNA changes could be secondary or adaptive responses to HFD. Future research should experimentally validate the predicted epi-miRNA–target interactions using functional assays (e.g., the luciferase reporter assay, expression analysis and cross-linking and immunoprecipitation (CLIP) assay). Time-course analyses and single-cell RNA sequencing would help clarify their mechanistic role and provide insights into the understanding of dynamic and context-dependent miRNA–target interactions. Gain- and loss-of-function experiments may elucidate the mechanistic pathways through which epi-miRNAs influence epigenetic regulation and contribute to cardiac pathology.

The identification of epi-miRNAs and their possible targets in HFD-induced cardiac dysfunction highlights potential therapeutic targets and epigenetic modulating strategies. For instance, RNA-based interventions (like antagonism or the mimicking of key epi-miRNAs), modulating EZH2 or inhibiting DNMTs may be promising in HFD-induced heart dysfunction. This study predicted increased DNA methylation and histone acetylation and decreased histone methylation in the hearts of HFD-fed rats. Therefore, the pharmacological inhibition of DNMTs and HAT activity may be promising in HFD-induced heart dysfunction. This speculation is in accordance with literature data showing the protective effect of DNMT inhibitors (e.g., RG108 [48], 5-azacytidine [49]) and HAT inhibitors (e.g., curcumin [56], L003 and C646 [57]) in heart failure or cardiac remodeling. Moreover, while in our study miRNA-132 upregulation due to HFD was predicted to modulate histone methylation and was associated with cardiac dysfunction, in recent clinical and preclinical studies, CDR132L, a synthetic oligonucleotide inhibitor of miRNA-132, showed beneficial effects in heart failure [58,59].

In conclusion, our research revealed putative epi-miRNAs and a complex epi-miRNA-mediated regulatory network in HFD-induced cardiac dysfunction. A comprehensive analysis of epi-miRNA–target interactions indicates increased DNA methylation and histone acetylation and decreased histone methylation in the hearts of HFD-fed rats. Our findings suggest the potential role of epi-miRNA-mediated pathological cardiac remodeling and dysfunction in obese rats fed with HFD and point to novel putative directions of potential pharmacological interventions in the future.

## 4. Materials and Methods

### 4.1. Cardiac miRNA Dataset of HFD-Fed Rats

Our in silico analysis was conducted on a previously published miRNA dataset on high-fat diet-induced cardiac remodeling [10]. In that study, 12-week-old Sprague Dawley rats were fed a high-fat diet (60% standard chow, 20% pork fat, 15% refined sugar, 1.5% cholesterol, 0.1% sodium cholate, 3.4% peanuts) or normal diet for 24 weeks. The total energy content of the high-fat diet and normal diet was 24.5% and 5% fat, respectively. At the end of the 24-week feeding period, the high-fat diet-fed rats showed significant obesity, myocardial fibrosis and impaired left ventricular function. A microarray analysis was performed on a part of the heart (*n* = 7 in each group) obtained at the end of the feeding period to assess miRNA expression profiles. miRNAs with a 2-fold or greater fold change and a *p*-value < 0.05 were considered differentially expressed (fold changes and *p* values are available as supplementary material of the original article [10]). A total of 94 miRNAs were differently expressed in the heart of high-fat diet-fed rats versus control; 50 were upregulated, and 44 were downregulated. The six most upregulated miRNAs (rno-let-7b-3p, rno-let-7c-5p, rno-let-7d-5p, rno-let-7f-1-3p, rno-miR-100-5p, rno-miR-10b-5p) were validated by qRT-PCR, indicating the good validity of the microarray results.

### 4.2. Target Prediction

Two independent databases were used for the bioinformatic prediction of miRNA targets, (i) the miRDB (https://mirdb.org/, version: 6.0) [60,61] and (ii) the mirWalk (http://mirwalk.umm.uni-heidelberg.de/search_mirnas/, version: 3.0) [62] databases.

miRDB uses the MirTarget support vector machine model, and it is able to perform transcriptome-wide miRNA predictions based on the miRBase version 22 and NCBI RefSeq databases [60,61]. miRDB ranks the predicted targets by assigning them a ‘target score’. These scores are given by the algorithm that calculates the prediction of the target. The higher the score, the more confident the prediction is. The maximum target score is 100. miRWalk uses the TarPmiR algorithm, which was developed to perform genome-wide miRNA target predictions [62]. The algorithm suggests the putative miRNA-binding sites within the complete sequence (promoter, CDS, 5′- and 3′-UTR regions) of all known genes. MirWalk ranks the predictions by a binding probability score. The binding probability is similar to the ‘target score’ in miRDB; it provides the confidence level of the predicted interaction, and its maximum value is 1.0.

All the predicted targets of each cardiac miRNA induced by HFD [10] with a ≥60 target score value and ≥0.8 binding probability were downloaded from miRDB and mirWalk, respectively. The high-threshold predictive limit was set to a ≥80 target score in miRDB and ≥0.92 binding probability in miRWalk. A cumulative prediction score was calculated as (miRDB target score/100) + miRWalk binding probability value.

### 4.3. Establishment of Epigenetic Regulators Expressed in the Heart

Genes with an epigenetic function annotated to the following Gene Ontology (GO) terms in the Rat Genome Database [63] were collected (retrieved 22 July 2024) and considered as epigenetic regulators: DNA-methyltransferase activity (GO:0009008), DNA demethylase activity (GO:0035514), histone acetyltransferase activity (GO:0004402), histone deacetylase activity (GO:0004407), histone methyltransferase activity (GO:0042054), histone demethylase activity (GO:0032452), histone ubiquitin ligase activity (GO:0140852), histone deubiquitinase activity (GO:0140934), histone kinase activity (GO:0035173), histone phosphatase activity (GO:0140789), histone succinyltransferase activity (GO:0106078), histone lactyltransferase activity (GO:0120301), histone glutaryltransferase activity (GO:0106229), histone butyryltransferase activity (GO:0140069), the positive regulation of gene expression via chromosomal CpG island demethylation (GO:0044029) and the epigenetic programming of male pronucleus (GO:0044727). According to the database, 248 epigenetic regulators expressed in the rat were identified based on the aforementioned GO terms. Since the cardiac miRNA dataset was gathered from adult rat hearts, these epigenetic regulators were further filtered to gain age- and heart-specific expressions. RNA-SEQ expression data of epigenetic regulator genes were retrieved from the Rat Genome Database [63] that provides transcriptomic profiles of gene expression across various tissues and developmental stages of rats. Only epigenetic regulators expressed in adult rat hearts were included in this computational study. Out of the 248 regulators, 219 epigenetic regulators showed a detectable expression in healthy adult heart tissue (Table S2).

To identify cardiac dysfunction- and remodeling-related epigenetic regulator targets, the Rat Genome Database disease ontology (DO) was used. Epigenetic regulators annotated to heart disease (DOID:114), ventricular dysfunction (DOID:900703), congestive heart failure (DOID:6000), cardiomyopathy (DOID:0050700) and cardiac fibrosis (DOID:9003139) were collected.

### 4.4. Identification of Epi-miRNAs

The cardiac miRNA dataset of HFD-fed rats, their predicted targets from miRDB and miRWalk and epigenetic regulators expressed in adult rat heart were loaded into a database management system (Microsoft Access, version: 2501). HFD-induced miRNAs targeting the same epigenetic regulators in both the miRDB and miRWalk prediction databases were identified as putative epi-miRNAs in this study (see Section 4.2). Putative epi-miRNAs reaching the high-threshold predicative limit indicate a higher confidence of modulating the predicted epigenetic regulator. A literature search was conducted to reveal miRNAs that were previously identified as epi-miRNAs in non-HFD animal or human studies.

### 4.5. Network Analysis

Each of the miRNA and predicated target pairs were imported into Cytoscape v3.9.0. [64] with the following data: HFD-induced miRNA name and direction of the change (down- or upregulation), the predicted target mRNA’s symbol and cumulative prediction score. Subsequently an miRNA–target network was constructed. Edges between the epi-miRNAs and target mRNA nodes were established according to the cumulative prediction score values. A cluster analysis was performed with clusterMaker2 v1.3.1 using the Markov Clustering Algorithm of Cytoscape. The clustering was based on the predicted score and calculated similarity score values between the nodes.

The STRING 12.0 (https://string-db.org/) [65] platform was used to analyze the interaction and enrichment of the predicted targets of putative epigenetic regulator epi-miRNAs. The minimum required interaction score was set to high confidence (0.700). MCL clustering was applied with inflation parameter 3. Local Network Cluster (STRING) and Biological Process (Gene Ontology) enrichment analyses were performed.

## Figures and Tables

**Table 1:** Putative cardiac epi-miRNAs and their predicted epigenetic regulator targets in high-fat diet (HFD)-induced cardiac dysfunction and remodeling. miRNAs changed in rat heart due to HFD are listed in alphabetical order. Putative epigenetic regulator targets, predicted by both miRDB and miRWalk databases, ordered according to cumulative prediction score (miRDB target score/100 + miRWalk binding probability). Blue background indicates epi-miRNAs that target regulators (indicated in bold–italic) with high-threshold predicative limits (i.e., ≥80 target score in miRDB and ≥0.92 binding probability in miRWalk). Y = yes; N = no.

**Upregulated Cardiac miRNAs in Response to HFD** [10] **and Their Predicted Epigenetic Regulator Targets**				
**miRNA Name**	**Predicted mRNA Target Symbol**	**Cumulative Prediction Score**	**Identified Previously as Epi-miRNA**	
			**Y/N**	**Target (*Species* *and* *Tissue*)**
**rno-let-7b-3p**	***Dcaf1*, *Kdm2b*, *Pcgf5*,** Clock, Dot1l	***1.95*, *1.91*, *1.89*,** 1.59, 1.48	N	-
**rno-let-7c-5p**	***Kdm3a*, *Mier1*,** Alkbh4	***1.73*, *1.73*,** 1.71	Y	Ezh2 (*mouse embryonic stem cells*) [20]
**rno-let-7f-1-3p**	***Kdm2b*** **, *Pcgf5***	***1.91*** **, *1.81***	N	-
**rno-miR-10b-5p**	Kdm5b	1.61	N	-
**rno-miR-126a-5p**	Setd5	1.54	Y	Dnmt1 (*human esophageal squamous cell*) [21]
**rno-miR-132-3p**	***Setd5*, *Kdm5a***, Setd7	***1.87*, *1.80,*** 1.46	Y	Sirt1 (*human proximal renal tubular epithelial cells*) [22] Hdac3 (*mouse neonatal cardiomyocytes*) [23]
**rno-miR-133a-3p**	Dot1l	1.63	Y	Ezh2 (*mouse neonatal cardiac fibroblasts*) [24]
**rno-miR-133b-3p**	Dot1l	1.71	Y	Dnmt3a (*mouse HL-1 cardiomyocytes*) [25]
**rno-miR-146b-3p**	***Setd5,*** Prkca, Kmt5c	***1.86,*** 1.61, 1.55	N	-
**rno-miR-148b-5p**	Kdm5a, Naa50	1.78, 1.64	N	-
**rno-miR-18a-3p**	Tada2a, Smyd2	1.69, 1.51	N	-
**rno-miR-217-3p**	Mecom, Prkcb, Rnf168, Kdm5a	1.70, 1.66, 1.58, 1.57	Y	Ehmt1, Ehmt2 (*rat neonatal cardiomyocytes*) [26]
**rno-miR-26a-5p**	***Usp3***, ***Ezh2,*** Ing3, Nsd2	***1.92*, *1.78,*** 1.62, 1.50	Y	Ezh2 (*rat neonatal cardiomyocytes*) [27]
**rno-miR-299b-5p**	***Bub1,*** Hdac4, Kdm4a, Rsbn1, Clock	***1.79,*** 1.71, 1.70, 1.69, 1.55	N	-
**rno-miR-322-5p**	***Ash1l*, *Clock,*** Usp49, Chek1, Rsbn1	***1.92*, *1.85,*** 1.68, 1.60, 1.56	N	-
**rno-miR-324-3p**	Prmt1	1.65	Y	Hdac6 (*human HeLa cells*) [28]
**rno-miR-330-5p**	Sirt2, Ehmt2, Jmjd6, Prkca	1.77, 1.71, 1.70, 1.54	N	-
**rno-miR-343**	***Nsd1*, *Kdm2a,*** Clock	***1.89*, *1.80,*** 1.64	N	-
**rno-miR-3547**	***Naa40***, Aurkb	***1.80,*** 1.76	N	-
**rno-miR-3572**	***Jmjd1c,*** Dcaf1, Naa50, Kat6a, Kmt5b	***1.86,*** 1.78, 1.76, 1.74, 1.66	N	-
**rno-miR-3583-3p**	***Prkcb,*** Kmt5b, Ing3, Clock	***1.93,*** 1.65, 1.60, 1.53	N	-
**rno-miR-3590-5p**	Naa50, Dtx3l	1.69, 1.67	N	-
**rno-miR-3593-3p**	Ash1l	1.70	N	-
**rno-miR-375-5p**	Pcgf5	1.69	N	-
**rno-miR-376c-5p**	Kat6a, Setd2	1.77, 1.74	N	-
**rno-miR-410-5p**	Setd5, Prkca	1.74, 1.73	N	-
**rno-miR-434-5p**	Atxn3	1.68	N	-
**rno-miR-484**	Prkcb, Clock, Ezh1	1.77, 1.73, 1.54	N	-
**rno-miR-674-3p**	***Alkbh4,*** Prkca, Usp49	***1.88,*** 1.78, 1.61	N	-
**rno-miR-741-3p**	Crebbp, Kdm5a, Kmt2e	1.68, 1.56, 1.53	N	-
**rno-miR-764-5p**	Naa50	1.71	N	-
**rno-miR-874-5p**	***Sirt3,*** Brpf1, Mta2, Kdm4a	***1.81,*** 1.79, 1.75, 1.66	N	-
**rno-miR-881-3p**	***Clock,*** Jmjd1c	***1.82,*** 1.60	N	-
**rno-miR-92b-3p**	***Usp36*, *Ezh2*, *Kmt5b*, *Atxn3*, *Rsbn1*, *Mier3***, Setd5	***1.94*, *1.90*, *1.89*, *1.88*, *1.85*, *1.75***, 1.74	Y	Prmt5 (*human B-lymphocytes*) [29]
**Downregulated Cardiac miRNAs in Response to HFD** [10] **and Their Predicted Epigenetic Regulator Targets**				
**miRNA Name**	**Predicted mRNA Target Symbol**	**Cumulative Prediction Score**	**Identified previously as epi-miRNA**	
			**Y/N**	**Target (*Species* *and* *Tissue*)**
**let-7d-5p**	Alkbh4, Mier1, Kdm3a	1.79, 1.79, 1.71	Y	Kdm3a (*human placental BeWo cells*) [11]; Tet2 (*mouse macrophages*) [30,31]
**rno-miR-100-5p**	Ntmt1	1.60	N	-
**rno-miR-1193-3p**	Naa50	1.69	N	-
**rno-miR-140-3p**	Kmt5b, Usp49, Kdm5a, Ing4, Mier3	1.76, 1.68, 1.66, 1.63, 1.62	N	-
**rno-miR-142-3p**	Clock, Ash1l	1.67, 1.64	N	-
**rno-miR-148b-3p**	***Nsd2***, Kat7, Mier1, Dnmt1	***1.79,*** 1.76, 1.68, 1.65	N	-
**rno-miR-183-3p**	***Kdm5b,*** Cdk2	***1.91,*** 1.65	N	-
**rno-miR-183-5p**	Kdm2b, Prkca, Baz1b	1.73, 1.71, 1.61	N	-
**rno-miR-1843a-5p**	***Phf8,*** Clock, Usp36, Naa60	***1.81,*** 1.61, 1.59, 1.51	N	-
**rno-miR-186-5p**	Rps6ka5, Mier1, Dcaf1	1.78, 1.77, 1.67	N	-
**rno-miR-193a-3p**	Kat5	1.66	N	-
**rno-miR-194-5p**	***Setd5,*** Rsbn1, Naa50, Clock	***1.81,*** 1.79, 1.65, 1.63	N	-
**rno-miR-195-5p**	***Clock,*** Rsbn1, Usp49, Ezh1, Chek1	***1.85,*** 1.66, 1.62, 1.61, 1.54	Y	Mbd1 (*mouse neural stem cells*) [32]
**rno-miR-199a-3p**	***Kdm3a,*** Dnmt3a	***1.87,*** 1.57	Y	Dnmt3a, Rap2a (*human papillary thyroid cancer cells*) [33]; Kdm3a (*rat mesenchymal stem cells*) [34]
**rno-miR-19b-3p**	***Mier3,*** Mier1, Mecom, Rps6ka5	***1.75,*** 1.72, 1.64, 1.61	N	-
**rno-miR-204-3p**	***Phf2***	***1.74***	N	-
**rno-miR-21-3p**	***Naa50*, *Setd2,*** Ing3, Setd5	***1.89*, *1.76,*** 1.69, 1.68	Y	Hdac8 (*human embryonic kidney 293 cells*) [35]
**rno-miR-22-3p**	***Phf8*, *Rsbn1,*** Usp36, Kdm6b, Kat6a	***1.92*, *1.81,*** 1.67, 1.64, 1.62	N	-
**rno-miR-297**	Clock	1.68	N	-
**rno-miR-29c-3p**	***Clock*, *Dnmt3a*, *Dot1l*, *Naa40,*** Kdm2a	***1.89*, *1.88*, *1.85*, *1.85*,** 1.76	Y	Dnmt3a, Tet1 (*human ovarian carcinoma*) [36]
**rno-miR-300-5p**	Dnmt1, Kdm3a, Taf1, Ccnb1	1.59, 1.58, 1.55, 1.55	N	-
**rno-miR-30e-3p**	***Atxn3,*** Prkaa1, Clock, Kdm5a	***1.86*,** 1.75, 1.54, 1.51	Y	Rps6kb1 (*human esophageal cancer cells*) [37]
**rno-miR-31a-5p**	***Wdr5, Rsbn1,*** Nsd3, Bap1, Clock, Ash1l	***1.88*, *1.79*, *1.74***, 1.69, 1.65, 1.62	N	-
**rno-miR-342-3p**	Kdm4a	1.76	Y	Dnmt1 (*human colorectal cancer SW480 cells*) [38]
**rno-miR-34c-5p**	Jmjd1c, Prkcb, Hr, Hdac1, Jade2	1.77, 1.74, 1.65, 1.61, 1.45	N	-
**rno-miR-3559-5p**	***Prkcb,*** Mier1, Rps6ka5	***1.88,*** 1.87, 1.66	N	-
**rno-miR-421-3p**	Kat6a	1.51	N	-
**rno-miR-466b-5p**	***Dnmt1,*** Crebbp	***1.81,*** 1.62	N	-
**rno-miR-496-5p**	Setd5, Prkca	1.74, 1.69	N	-
**rno-miR-505-5p**	Alkbh4, Kat7, Rnf168	1.74, 1.68, 1.45	N	-
**rno-miR-653-3p**	***Ezh2,*** Dnmt1	***1.91,*** 1.58	N	-
**rno-miR-664-1-5p**	***Usp49,*** Clock	***1.81,*** 1.56	N	-
**rno-miR-664-2-5p**	***Usp49,*** Clock	***1.81,*** 1.64	N	-
**rno-miR-667-5p**	***Dnmt3a*, *Prdm2,*** Carm1, Mier2	***1.85*, *1.78,*** 1.76, 1.49	N	-
**rno-miR-7a-5p**	***Ezh1*, *Prkcb,*** Sirt5, Jade1, Smyd5	***1.82*, *1.76,*** 1.65, 1.59, 1.55	N	-
**rno-miR-872-5p**	Dtx3l, Kat6a	1.69, 1.62	N	-
**rno-miR-874-3p**	***Kdm4a***	***1.81***	N	-

**Table 2:** Predicted epigenetic regulator targets of epi-miRNAs in high-fat diet (HFD) induced cardiac dysfunction and remodeling. The putative epigenetic regulator targets of cardiac miRNAs, predicted by both the miRDB and miRWalk databases, are listed in the table. A few miRNAs have alternative symbols, more relevant to epigenetic regulation; those are indicated in brackets after the symbol. Targets grouped according to their epigenetic function. Gene Ontology (GO) molecular function accession numbers indicated in brackets after the function. Heart expression levels of the predicated targets are based on RNA-SEQ expression data published in the Rat Genome Database: medium 11–1000 TPM, low 0.5–10 TPM. Relevant heart disease annotations to HFD-induced cardiac dysfunction are in bold. Evidence codes represent inferred from expression pattern (IEP), from sequence orthology (ISO) and from sequence or structural similarity (ISS). Red background shows a target with only upregulated epi-miRNAs; green background shows a target with only downregulated epi-miRNAs.

**Predicted mRNA Target**					**Number of Epi-miRNAs Induced by HFD [10] That Target the Predicted mRNA**		
**Symbol** **(Alternative Symbol)**	**Name**	**Gene ID**	**Expression Level in Heart**	**Annotated to Heart Disease (Evidence Code)**	**Total**	**Up-Regulated**	**Down-Regulated**
**DNA-methyltransferase activity (GO:0009008)**							
Dnmt1	DNA methyltransferase 1	84350	low	**congestive heart failure (IEP), cardiomyopathy (ISO)**	4	0	4
Dnmt3a	DNA methyltransferase 3 alpha	444984	low	congenital heart disease (IEP)	3	0	3
**DNA demethylase activity (GO:0035514)**							
Alkbh4	alkB homolog 4, lysine demethylase	288587	low	NO	4	2	2
**histone acetyltransferase activity (GO:0004402)**							
Brpf1	bromodomain and PHD finger containing, 1	679713	low	**cardiomyopathy (ISO)**	1	1	0
Clock (Kat13d)	clock circadian regulator	60447	medium	NO	17	7	10
Crebbp (Kat3a)	CREB binding protein	54244	medium	**cardiomyopathy (IEP)**	2	1	1
Ing3	inhibitor of growth family, member 3	312154	low	NO	3	2	1
Ing4	inhibitor of growth family, member 4	297597	medium	NO	1	0	1
Jade1	jade family PHD finger 1	310352	medium	NO	1	0	1
Jade2	jade family PHD finger 2	303113	low	NO	1	0	1
Kat5	lysine acetyltransferase 5	192218	medium	NO	1	0	1
Kat6a	lysine acetyltransferase 6A	306571	medium	congenital heart disease (ISS)	5	2	3
Kat7	lysine acetyltransferase 7	303470	medium	NO	2	0	2
Naa40 (Nat11)	N(alpha)-acetyltransferase 40, NatD catalytic subunit	361718	medium	NO	2	1	1
Naa50 (Nat5, Nat13, Mak3)	N(alpha)-acetyltransferase 50, NatE catalytic subunit	288108	medium	NO	7	4	3
Naa60 (Hat4)	N(alpha)-acetyltransferase 60, NatF catalytic subunit	363545	medium	NO	1	0	1
Tada2a	transcriptional adaptor 2A	360581	low	NO	1	1	0
Taf1 (Kat4)	TATA-box binding protein associated factor 1	317256	low	congenital heart disease (ISO)	1	0	1
**histone deacetylase activity (GO:0004407)**							
Atxn3	ataxin 3	60331	low	NO	3	2	1
Hdac1	histone deacetylase 1	297893	medium	**congestive heart failure (ISO)**	1	0	1
Hdac4	histone deacetylase 4	363287	low	**congestive heart failure (ISO)**	1	1	0
Mier1	MIER1 transcriptional regulator	313418	medium	NO	6	1	5
Mier2	MIER family member 2	362841	low	NO	1	0	1
Mier3	MIER family member 3	310086	low	NO	3	1	2
Mta2	metastasis associated 1 family, member 2	361724	medium	NO	1	1	0
Sirt2	sirtuin 2	361532	medium	NO	1	1	0
Sirt3	sirtuin 3	293615	medium	**congestive heart failure (IEP)**	1	1	0
Sirt5	sirtuin 5	306840	medium	NO	1	0	1
**histone methyltransferase activity (GO:0042054)**							
Ash1l (Kmt2h)	ASH1 like histone lysine methyltransferase	310638	medium	NO	4	2	2
Carm1	coactivator-associated arginine methyltransferase 1	363026	medium	NO	1	0	1
Dot1l (Kmt4)	DOT1 like histone lysine methyltransferase	362831	low	**cardiomyopathy (ISS)**	4	3	1
Ehmt2 (Kmt1c)	euchromatic histone lysine methyltransferase 2	361798	medium	NO	1	1	0
Ezh1 (Kmt6b)	enhancer of zeste 1 polycomb repressive complex 2 subunit	303547	medium	NO	3	1	2
Ezh2 (Kmt6a)	enhancer of zeste 2 polycomb repressive complex 2 subunit	312299	low	**congestive heart failure (ISO)**	3	2	1
Kmt2e	lysine methyltransferase 2E	311968	medium	NO	1	1	0
Kmt5b	lysine methyltransferase 5B	361688	medium	NO	4	3	1
Kmt5c	lysine methyltransferase 5C	308345	medium	NO	1	1	0
Mecom (Kmt8e)	MDS1 and EVI1 complex locus	294924	low	NO	2	1	1
Nsd1 (Kmt3b)	nuclear receptor binding SET domain protein 1	306764	medium	congenital heart disease (ISO)	1	1	0
Nsd2 (Kmt3g)	nuclear receptor binding SET domain protein 2	680537	low	**cardiomyopathy (ISO)**	2	1	1
Nsd3 (Kmt3f)	nuclear receptor binding SET domain protein 3	290831	low	NO	1	0	1
Ntmt1	N-terminal Xaa-Pro-Lys N-methyltransferase 1	362103	medium	NO	1	0	1
Prdm2 (Kmt8, Kmt8a)	PR/SET domain 2	313678	low	NO	1	0	1
Prmt1	protein arginine methyltransferase 1	60421	medium	NO	1	1	0
Setd2 (Kmt3a)	SET domain containing 2, histone lysine methyltransferase	316013	medium	NO	2	1	1
Setd5	SET domain containing 5	297514	medium	**cardiomyopathy (ISO)**	8	5	3
Setd7 (Kmt7)	SET domain containing 7, histone lysine methyltransferase	689954	medium	NO	1	1	0
Smyd2 (Kmt3c)	SET and MYND domain containing 2	289372	medium	NO	1	1	0
Smyd5	SMYD family member 5	312503	low	NO	1	0	1
Wdr5	WD repeat domain 5	362093	medium	congenital heart disease (ISO)	1	0	1
**histone demethylase activity (GO:0032452)**							
Hr	HR, lysine demethylase and nuclear receptor corepressor	60563	low	congenital heart disease (ISO)	1	0	1
Jmjd1c (Kdm3c)	jumonji domain containing 1C	171120	medium	NO	3	2	1
Jmjd6	jumonji domain containing 6, arginine demethylase and lysine hydroxylase	360665	medium	NO	1	1	0
Kdm2a	lysine demethylase 2A	361700	medium	**congestive heart failure (ISO)**	2	1	1
Kdm2b	lysine demethylase 2B	304495	low	NO	3	2	1
Kdm3a	lysine demethylase 3A	312440	medium	NO	4	1	3
Kdm4a	lysine demethylase 4A	313539	medium	**cardiomegaly (ISO)**	4	2	2
Kdm5a	lysine demethylase 5A	312678	medium	NO	6	4	2
Kdm5b	lysine demethylase 5B	304809	low	myocardial infarction (ISO)	2	1	1
Kdm6b	lysine demethylase 6B	363630	medium	NO	1	0	1
Phf2 (Kdm7c)	PHD finger protein 2	306814	medium	NO	1	0	1
Phf8 (Kdm7b)	PHD finger protein 8	317425	low	NO	2	0	2
Rsbn1 (Kdm9)	round spermatid basic protein 1	310749	low	NO	7	3	4
**histone ubiquitin ligase activity (GO:0140852)**							
Dtx3l	deltex E3 ubiquitin ligase 3L	498089	low	NO	2	1	1
Pcgf5	polycomb group ring finger 5	681178	medium	NO	3	3	0
Rnf168	ring finger protein 168	690043	low	NO	2	1	1
**histone deubiquitinase activity (GO:0140934)**							
Bap1	Brca1 associated protein 1	306257	medium	**cardiomyopathy (ISO)**	1	0	1
Usp3	ubiquitin specific peptidase 3	363084	low	**cardiomyopathy (ISO)**	1	1	0
Usp36	ubiquitin specific peptidase 36	303700	low	NO	3	1	2
Usp49	ubiquitin specific peptidase 49	316211	low	NO	6	2	4
**histone kinase activity (GO:0035173)**							
Aurkb	aurora kinase B	114592	low	NO	1	1	0
Baz1b	bromodomain adjacent to zinc finger domain, 1B	368002	medium	Williams–Beuren syndrome (ISO)	1	0	1
Bub1	BUB1 mitotic checkpoint serine/threonine kinase	296137	low	NO	1	1	0
Ccnb1	cyclin B1	25203	low	NO	1	0	1
Cdk2	cyclin dependent kinase 2	362817	low	NO	1	0	1
Chek1	checkpoint kinase 1	140583	low	NO	2	1	1
Dcaf1	DDB1 and CUL4 associated factor 1	315987	low	NO	3	2	1
Prkaa1 (Ampka1)	protein kinase AMP-activated catalytic subunit alpha 1	65248	low	NO	1	0	1
Prkca (Pkca, Pkcaalpha, Pkcα)	protein kinase C, alpha	24680	low	**congestive heart failure (IEP), cardiomyopathy (ISO)**	6	4	2
Prkcb (Pkcb, Pkcbeta, Pkcβ)	protein kinase C, beta	25023	low	**congestive heart failure (IEP), cardiomyopathy (ISO)**	6	3	3
Rps6ka5 (Msk1)	ribosomal protein S6 kinase A5	314384	low	NO	3	0	3

## Data Availability

All the data generated in this publication are available upon request to the authors.

## Supplementary Materials

The following supporting information can be downloaded at: https://www.mdpi.com/article/10.3390/ijms26052247/s1. References [66,67,68,69,70,71,72,73,74,75] are cited in the supplementary materials.

## Abbreviations

The following abbreviations are used in this manuscript:

Dnmt	DNA methyltransferase
epi-miRNA	Epigenetic Regulator microRNAs
Ezh2	Enhancer of Zeste homolog 2
Hat	Histone acetyltransferase
Hdac	Histone deacetylase
HFD	High-fat diet
miRNA	Micro RNA
Myh7	β-myosin heavy chain
