# Supplementary material for: Putative Epigenetic Regulator microRNAs (epi-miRNAs) and Their Predicted Targets in High-Fat Diet-Induced Cardiac Dysfunction: An In Silico Analysis in Obese Rats
Source: Int J Mol Sci. 2025 Mar 3;26(5):2247. doi: 10.3390/ijms26052247 (PMC11900980; doi:10.3390/ijms26052247)
Supplement: Supplementary file 1 [file ijms-26-02247-s001.zip › TableS1.pdf]

Table S1. Literature search on validation of epi-miRNAs-target interactions that reached the high-threshold predictive limit in HFD-induced cardiac dysfunction.

miRNA Name	mRNA Symbol (predicted target)	Alternative symbol	Validated target? (Y/N)	Reference
miR-31a-5p	Wdr5	--	Y	[1, 2]
miR-664-1-5p	Usp49	--	N	
miR-664-2-5p	Usp49	--	N	
miR-92b-3p	Usp36	--	N	
miR-132-3p	Setd5	--	N	
miR-146b-3p	Setd5	--	N	
miR-22-3p	Rsbn1	Kdm9	N	
miR-3583-3p	Prkcb	PKCB, Pkcbeta, Pkcβ	N	
miR-22-3p	Phf8	Kdm7b	Y	[3, 4]
miR-1843a-5p	Phf8	Kdm7b	N	
let-7b-3p	Pcgf5	--	N	
miR-343	Nsd1	Kmt3b	N	
miR-29c-3p	Naa40	Nat11	N	
miR-3547	Naa40	Nat11	N	
miR-3559-5p	Mier1	--	N	
miR-132-3p	Kdm5a	Rbp2, **Jarid1a**	Y	[5]
miR-874-3p	Kdm4a	--	N	
miR-199a-3p	Kdm3a	--	Y	[6]
miR-653-3p	Ezh2	Kmt6a	N	
miR-92b-3p	Ezh2	Kmt6a	Y	[7]
miR-7a-5p	Ezh1	Kmt6b	N	
miR-29c-3p	Dot1l	Kmt4	N	
let-7b-3p	Dcaf1	--	N	
miR-29c-3p	Clock	Kat13d	N	
miR-195-5p	Clock	Kat13d	N	
miR-322-5p	Clock	Kat13d	N	
miR-881-3p	Clock	Kat13d	N	
miR-30e-3p	Atxn3	--	N	
miR-322-5p	Ash1l	Kmt2h	N	
miR-674-3p	Alkbh4	--	N	
miR-26a-5p	Usp3	--	Y	[8]
miR-667-5p	Dnmt3a	--	N	
miR-343	Kdm2a	--	N	
miR-874-5p	Sirt3	--	Y	[9]
miR-194-5p	Setd5	--	N	
miR-21-3p	Setd2	Kmt3a	N	
miR-92b-3p	Rsbn1	Kdm9	N	
miR-31a-5p	Rsbn1	Kdm9	N	
miR-3559-5p	Prkcb	PKCB, Pkcbeta, Pkcβ	N	
miR-7a-5p	Prkcb	PKCB, Pkcbeta, Pkcβ	Y	[10]
miR-674-3p	Prkca	PKCA, Pkcalpha, Pkcα	N	

miR-667-5p	Prdm2	Kmt8, Kmt8a	N	
miR-204-3p	Phf2	Kdm7c	N	
let-7f-1-3p	Pcgf5	--	N	
miR-148b-3p	Nsd2	Kmt3g	N	
miR-21-3p	Naa50	Nat5, Nat13, Mak3	N	
miR-92b-3p	Mier3	--	N	
let-7c-5p	Mier1	--	N	
miR-19b-3p	Mier1	--	N	
miR-92b-3p	Kmt5b	--	N	
miR-183-3p	Kdm5b	--	N	
let-7c-5p	Kdm3a	--	N	
let-7b-3p	Kdm2b	--	N	
let-7f-1-3p	Kdm2b	--	N	
miR-3572	Jmjd1c	Kdm3c	N	
miR-26a-5p	Ezh2	Kmt6a	Y	[11]
miR-29c-3p	Dnmt3a	--	Y	[12]
miR-466b-5p	Dnmt1	--	N	
miR-299b-5p	Bub1	--	N	
miR-92b-3p	Atxn3	--	N	

1. Ren, J., Intermittent hypoxia BMSCs-derived exosomal miR-31-5p promotes lung adenocarcinoma development via WDR5-induced epithelial mesenchymal transition *Sleep and Breathing* **2023,** 27, (4), 1399-1409.
2. Wu, J.; Tan, X.; Lin, J.; Yuan, L.; Chen, J.; Qiu, L.; Huang, W., Minicircle-oriP-miR-31 as a Novel EBNA1-Specific miRNA Therapy Approach for Nasopharyngeal Carcinoma. *Human Gene Therapy* **2016,** 28, (5), 415-427.
3. Cai, M.-Z.; Wen, S.-Y.; Wang, X.-J.; Liu, Y.; Liang, H., MYC Regulates PHF8, Which Promotes the Progression of Gastric Cancer by Suppressing miR-22-3p. *Technology in cancer research & treatment* **2020,** 19, 1533033820967472.
4. Shao, P.; Liu, Q.; Maina, P. K.; Cui, J.; Bair, T. B.; Li, T.; Umesalma, S.; Zhang, W.; Qi, H. H., Histone demethylase PHF8 promotes epithelial to mesenchymal transition and breast tumorigenesis. *Nucleic Acids Res* **2017,** 45, (4), 1687-1702.
5. Alvarez-Saavedra, M.; Antoun, G.; Yanagiya, A.; Oliva-Hernandez, R.; Cornejo-Palma, D.; Perez-Iratxeta, C.; Sonenberg, N.; Cheng, H.-Y. M., miRNA-132 orchestrates chromatin remodeling and translational control of the circadian clock. *Hum Mol Genet* **2011,** 20, (4), 731-751.
6. Wu, J.-C.; Sun, J.; Xu, J.-C.; Zhou, Z.-Y.; Zhang, Y.-F., Down-regulated microRNA-199a-3p enhances osteogenic differentiation of bone marrow mesenchymal stem cells by targeting Kdm3a in ovariectomized rats. *Biochemical Journal* **2021,** 478, (4), 721-734.
7. Liu, F.; Sang, M.; Meng, L.; Gu, L.; Liu, S.; Li, J.; Geng, C., miR-92b promotes autophagy and suppresses viability and invasion in breast cancer by targeting EZH2. *Int J Oncol* **2018,** 53, (4), 1505-1515.
8. Gao, S.; Li, J.; Song, L.; Wu, J.; Huang, W., Influenza A virus-induced downregulation of miR-26a contributes to reduced IFNα/β production. *Virologica Sinica* **2017,** 32, (4), 261-270.
9. Zhang, L.; Ma, C.; Wang, X.; Bai, J.; He, S.; Zhang, J.; Xin, W.; Li, Y.; Jiang, Y.; Li, J.; Zhu, D., MicroRNA-874-5p regulates autophagy and proliferation in pulmonary artery smooth muscle cells by targeting Sirtuin 3. *European Journal of Pharmacology* **2020,** 888, 173485.

10. Latreille, M.; Hausser, J.; Stützer, I.; Zhang, Q.; Hastoy, B.; Gargani, S.; Kerr-Conte, J.; Pattou, F.; Zavolan, M.; Esguerra, J. L.; Eliasson, L.; Rülicke, T.; Rorsman, P.; Stoffel, M., MicroRNA-7a regulates pancreatic β cell function. *J Clin Invest* **2014,** 124, (6), 2722-35.
11. Chen, M.; Lin, Y.; Guo, W.; Chen, L., BMSC-Derived Exosomes Carrying miR-26a-5p Ameliorate Spinal Cord Injury via Negatively Regulating EZH2 and Activating the BDNF-TrkB-CREB Signaling. *Mol Neurobiol* **2024,** 61, (10), 8156-8174.
12. Zhao, H.; Feng, L.; Cheng, R.; Wu, M.; Bai, X.; Fan, L.; Liu, Y., miR-29c-3p acts as a tumor promoter by regulating β-catenin signaling through suppressing DNMT3A, TET1 and HBP1 in ovarian carcinoma. *Cellular Signalling* **2024,** 113, 110936.
