# Supplementary figures and images for: Putative Epigenetic Regulator microRNAs (epi-miRNAs) and Their Predicted Targets in High-Fat Diet-Induced Cardiac Dysfunction: An In Silico Analysis in Obese Rats
Source: Int J Mol Sci. 2025 Mar 3;26(5):2247. doi: 10.3390/ijms26052247 (PMC11900980; doi:10.3390/ijms26052247)

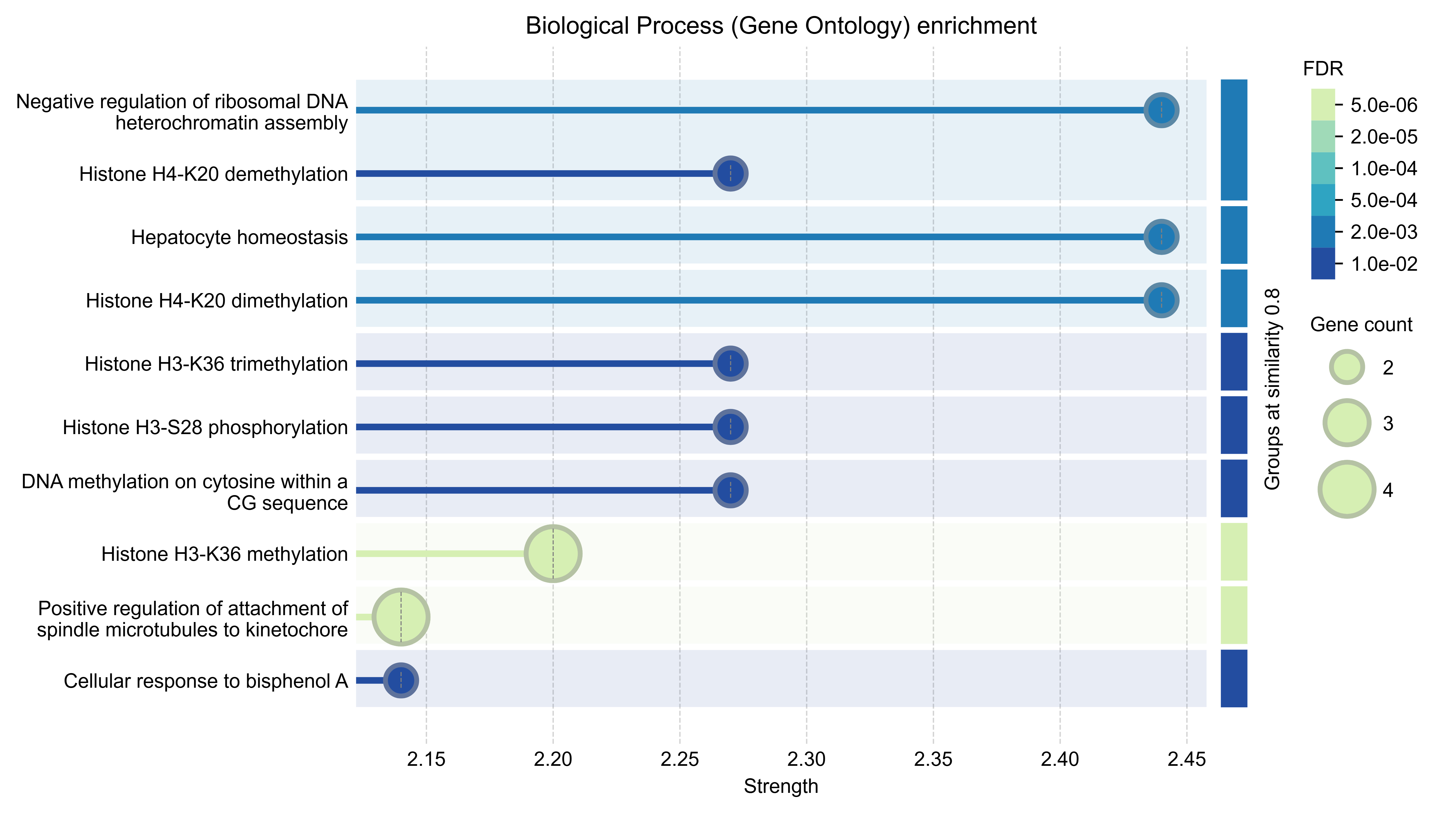

Supplement: Supplementary file 1 [file ijms-26-02247-s001.zip › FigureS1.png]
